# A Pilot Project to Promote Research Competency in Medical Students Through Journal Clubs: Mixed Methods Study

**DOI:** 10.2196/51173

**Published:** 2024-10-31

**Authors:** Mert Karabacak, Zeynep Ozcan, Burak Berksu Ozkara, Zeynep Sude Furkan, Sotirios Bisdas

**Affiliations:** 1 Department of Neurosurgery, Mount Sinai Health System, New York, NY, United States; 2 Cerrahpasa Faculty of Medicine, Istanbul University—Cerrahpasa, Istanbul, Turkey; 3 Department of Neuroradiology, MD Anderson Cancer Center, Houston, TX, United States; 4 Lysholm Department of Neuroradiology, The National Hospital for Neurology and Neurosurgery, University College London Hospitals NHS Foundation Trust, Neuroimaging Analysis Centre, Queen Square 8-11, London, WC1N3BG, United Kingdom, 44 2034483446

**Keywords:** medical student, research, peer education, student society, journal club, skills, scientific investigation, undergraduate, student-led, initiative, resources, research training, competency, continuing education, research improvement, motivation, mentor, mentorship, medical education

## Abstract

**Background:**

Undergraduate medical students often lack hands-on research experience and fundamental scientific research skills, limiting their exposure to the practical aspects of scientific investigation. The Cerrahpasa Neuroscience Society introduced a program to address this deficiency and facilitate student-led research.

**Objective:**

The primary goal of this initiative was to enhance medical students’ research output by enabling them to generate and publish peer-reviewed papers within the framework of this pilot project. The project aimed to provide an accessible, global model for research training through structured journal clubs, mentorship from experienced peers, and resource access.

**Methods:**

In January 2022, a total of 30 volunteer students from various Turkish medical schools participated in this course-based undergraduate research experience program. Students self-organized into 2 groups according to their preferred study type: original research or systematic review. Two final-year students with prior research experience led the project, developing training modules using selected materials. The project was implemented entirely online, with participants completing training modules before using their newly acquired theoretical knowledge to perform assigned tasks.

**Results:**

Based on student feedback, the project timeline was adjusted to allow for greater flexibility in meeting deadlines. Despite these adjustments, participants successfully completed their tasks, applying the theoretical knowledge they had gained to their respective assignments. As of April 2024, the initiative has culminated in 3 published papers and 3 more under peer review. The project has also seen an increase in student interest in further involvement and self-paced learning.

**Conclusions:**

This initiative leverages globally accessible resources for research training, effectively fostering research competency among participants. It has successfully demonstrated the potential for undergraduates to contribute to medical research output and paved the way for a self-sustaining, student-led research program. Despite some logistical challenges, the project provided valuable insights for future implementations, showcasing the potential for students to engage in meaningful, publishable research.

## Introduction

Undergraduate medical students frequently face limited opportunities for hands-on research experience [1,2]. Current medical school curricula often fail to equip students adequately with fundamental scientific research skills. Despite a high proportion of students expressing interest in research, only a small fraction possesses a thorough understanding of the medical research process [3]. In addition, empirical evidence underlines the contribution of undergraduate research engagement to career progression in medicine [4]. Consequently, medical students, cognizant of research’s significance, are increasingly seeking opportunities to augment their involvement [5]. To facilitate this quest, various course-based undergraduate research experience (CURE) programs have emerged, albeit with room for further refinement [6].

The emergence of remote learning, coupled with the proliferation of web-based platforms and open-access journals, has amplified data accessibility and the availability of research tools. This shift has catalyzed scientific literacy development and enabled self-paced learning among students across diverse disciplines. Students can now opt for extracurricular web-based courses or access materials of varying media to deepen their understanding of selected topics, acquire new skills, and enhance their overall capabilities. Beyond an individualistic approach, web-based platforms have simplified the process for students to find groups for information exchange, thereby bolstering their scientific understanding. The Cerrahpasa Neuroscience Society, a student-led organization, hosts 4 journal clubs wherein students gather online to discuss neurosurgery, neurology, psychiatry, and neuroscience through selected papers and subsequent discussions [7]. The primary aim of this research initiative is to stimulate and mentor students within these journal clubs to undertake their own research projects, leveraging a structured program replete with experienced near-peer guidance and comprehensive information access. The program’s ultimate objective is the inception and publication of fully student-run studies and papers in peer-reviewed journals, marrying theoretical knowledge with research fundamentals in a hands-on setting.

Our organizing team, composed of 1 second-year and 2 final-year medical students (MK, ZO, and BBO), sought to exploit the omnipresence of information and the scientific curiosity of journal club participants. We embarked on a pilot project with the clear ambition of significantly enhancing the research output of undergraduate medical students.

## Methods

### Ethical Considerations

Ethical approval was deemed unnecessary by the Istanbul University-Cerrahpasa — Cerrahapasa Faculty of Medicine Institutional Review Board as the survey responses were anonymous and participants consented to their data being used for research purposes. Participants’ data were anonymized and no compensation was provided for the participants. In addition, the data originated from the activities of the Cerrahpasa Neuroscience Society, which were conducted remotely and independently of the university.

### Planning

The project involved participants exclusively from the journal clubs, encompassing undergraduate medical students from various Turkish medical faculties and academic levels. We presented the project idea to all club members in December 2021, with 30 of the 40 members volunteering to partake in the project.

As the organizing committee, we compiled a series of introductory papers and vetted web-based courses centered around research fundamentals, which we arranged into scheduled training modules (Multimedia Appendices 1 and 2). We slated monthly briefings to guide and track participants’ progress, consistent with the project timeline. The final-year students in the organizing team (hereafter referred to as “the tutors”), who had accrued prior research experience, pinpointed research topics appropriate for undergraduate projects (Table 1).

**Table 1.** Study types and topics selected for research project implementation.

Journal club subject and study type		Research topic
**Neurosurgery**		
	Systematic Review	Medulloblastoma subgroup classification with radiomics
	Systematic Review	Radiosensitizing agents in medulloblastoma
**Neurology**		
	Original Study	Differentiation of SPMS^a^ formation using machine learning
	Systematic Review	The concurrence of multiple sclerosis and glioblastoma
**Psychiatry**		
	Original Study	Medical student stress, burnout, and depression in Turkey
	Original Study	Substance use and mental health among medical students in Turkey
**Neuroscience**		
	Original Study	Adolescents’ sleep and academic standing
	Systematic Review	Neuropsychological outcomes following radiation therapy of pediatric posterior fossa tumors

a SPMS: secondary progressive multiple sclerosis.

Study type selection was based on practical considerations and journal club subjects. Two systematic reviews were assigned to the neurosurgery journal club, as conducting original studies in this field would pose challenges for undergraduate students. The psychiatry journal club was tasked with creating 2 original studies using survey methods, whereas the neurology and neuroscience journal clubs were each assigned 1 systematic review and 1 original study. The project was planned to be entirely online, spanning 6 months from January 2022 to June 2022.

### Implementation

In January 2022, participants from the 4 journal clubs were segregated into 2 groups based on their chosen study type: original papers or systematic reviews, resulting in 8 project groups. Each group, composed of 3‐5 students, incorporated journal club participants and the clubs’ tutors. The project was guided by academic supervisors, who ensured methodological rigor and provided expert advice on the research topics. Tutors BBO and MK, both final-year medical students with prior research experience, actively identified suitable research questions, led regular web-based meetings, provided ongoing feedback, responded to participants’ queries, and facilitated navigation through the various stages of the research projects. Communication within these groups was facilitated through web-based chat platforms, with the organizing team included.

The educational materials for the project were meticulously selected based on the tutors’ personal experiences and an extensive review of available web-based resources. This process ensured that the materials were both relevant and of high educational quality. Following their initial briefing on project fundamentals and expectations, participants embarked on the first training module intended to acquaint them with research basics (Multimedia Appendix 1). This module contained a video series on using PubMed and Zotero (Center for History and New Media at George Mason University), along with 6 papers detailing the general steps of a research project. Participants were encouraged to complete these materials at their own pace within a 1-month time frame.

In February 2022, we classified tasks into 3 categories: “literature review and data extraction,” “statistical analysis,” and “manuscript writing.” Participants were allocated these tasks primarily based on their skills and interests. The second training module offered specific web-based courses for these tasks (Multimedia Appendix 2). Unlike the first module, the deadline for the second module was tailored to each participant’s task timeline. During monthly web-based briefings, the tutors provided assistance and feedback while illustrating task examples. Furthermore, tutors guided participants in ancillary tasks such as database access and ethics committee application form preparation.

Unexpected constraints led us to revise some study designs and the schedule. Two projects—a systematic review and an original study requiring database access—were discontinued and substituted with bibliometric analysis projects, supplemented by additional peer training. Heeding participant feedback, we also extended the original deadlines.

Bibliometric studies necessitated unique procedures, executed throughout March and April 2022 with regular briefings. In May 2022, a tutor conducted an auxiliary academic writing workshop. Although not every participant was tasked with study documentation, we believed all could glean valuable insights from this near-peer workshop within the project’s ambit. This workshop was made available to all participants, with a recorded version disseminated for those unable to attend the live session.

## Results

The project began with 30 undergraduate medical students, some contributing to multiple projects, and concluded with 25 participants successfully adhering to the full schedule. Those who withdrew from the project did so during the implementation of the second module, necessitating adjustments in task assignments and study configurations. The 25 students successfully adhering to the full schedule were from 5 universities, with a significant concentration (18 students) at Istanbul University—Cerrahpasa. The remainder was distributed to 4 other universities. Ten of these participants were enrolled in English language medical programs. The cohort consisted of 19 preclinical students, who were primarily enrolled in basic medical sciences courses, and 6 clinical students, who were completing clerkships and internships. The group included 14 female and 11 male students. None of the students had previous research experience.

The completion of the first module was gauged through participants’ feedback on the materials and their demonstrated proficiency in operating the platforms integrated into the module. Given that the second module encompassed verified web-based courses, completion was monitored via certifications from the respective platforms. As this module required the practical application of learned theoretical skills, successful task execution within the research study denoted each participant’s successful project completion.

Participants tasked with “literature review and data collection” and “statistical analysis” adeptly applied their theoretical knowledge acquired from the courses and briefings, creating necessary data tables and thus fulfilling their tasks. Owing to requisite timeline adjustments, those delegated to academic writing courses completed their tasks at disparate times relative to the original schedule. Nevertheless, manuscript creation for all studies was achieved, signifying that all participants made their respective contributions to the project.

During the implementation phase, we made some timeline alterations in response to student feedback, with participants requesting more accommodating deadlines. The tutors, who had previously conducted independent research projects, provided substantial support throughout the project’s execution. They shared their experiences and offered guidance, aiding participants in gaining a deeper understanding of their tasks. Upon the conclusion of second module training, participants shared feedback on the project’s implementation. As of April 2024, 3 papers have been published in peer-reviewed journals [8-10], and 4 papers have been submitted for peer review.

## Discussion

### Principal Findings

A multi-institutional study revealed that although 83% of the students surveyed believed that participating in research was educationally beneficial, only 31% thought that there was enough time allocated for it. In addition, just 15% felt that they received adequate training in research methodology, and only 25% considered the training in critical appraisal to be sufficient [11]. CUREs may help alleviate some of these issues, potentially improving access to both time and quality instruction in research skills. In parallel, CUREs have been adopted in universities to inspire students to pursue research, although these programs predominantly involve student participation in laboratory settings for data collection [6]. Efforts to enhance student contributions to research include a microbiology research initiative at a Canadian university aimed at publishing student-authored papers [12] and remote CUREs focusing on ecology at a US university [13]. However, our program offers a distinct approach.

The pilot project’s foremost insight is the successful operation of a fully student-run research program, from the training process to paper publication, using a method that is universally applicable to students interested in research. Instead of creating new lectures, we leveraged preexisting, verified web-based resources on research basics for our training modules. This strategy emphasizes the global accessibility of research training for students, as these resources are publicly available and facilitate self-paced learning. Notably, our research initiative aimed to exploit information accessibility not just only for skill acquisition but also for data collection to complete a research project. As undergraduate medical students have limited access to hands-on research, effectively using available information is crucial. To underscore global applicability and facilitate research involvement, the data used were sourced from 2 web-based platforms: academic literature and web-based surveys. Our project enabled the conduct of both systematic reviews and original studies by students lacking prior hands-on research experience, thereby enhancing student research output through remote involvement.

Regarding skill development, the project achieved the anticipated results. All participants completed their respective tasks, culminating in the production of completed manuscripts. This outcome demonstrates a tangible enhancement in participants’ research skills, particularly considering their nonexistent prior research experience. It is critical to note that participants did not receive identical training; instead, a division of labor was used. Participants learned about and practiced various aspects of research study design, with the project’s methodology allowing them to hone their skills in their chosen task within a study. As an immediate benefit, some participants expressed interest in completing the remaining web-based courses to further their skills, suggesting potential for project continuation. Feedback indicated a keen interest in furthering the concept. For instance, students initially assigned to work with databases expressed a desire to design and conduct a study upon completing their current work. Previous research suggests that students gain an improved understanding of the benefits of research following participation, and our project participants’ enthusiasm supports this finding [14].

A key success factor was the experienced near-peer tutoring that accompanied the project’s full duration. The final-year students on the organizing team designed the project outline, selected suitable research questions, monitored progress, and provided guidance as needed. Monthly briefings fostered an environment where participants reported progress and posed questions. Within these meetings, tutors also demonstrated tasks to facilitate student understanding. Participants gained comprehensive insight into the research process by first completing a course, then practicing an example task, and finally executing their respective tasks independently. Participant feedback suggested that near-peer tutoring facilitated question asking, contributing to a comfortable learning environment. Overall, including student guidance in the initiative increased the efficiency of the modules, as also affirmed by participants.

A significant advantage of the project’s design is its potential for self-sustainability. The students trained during this pilot project now have the experience to guide subsequent student cohorts looking to enhance their research skills. They can also offer fresh ideas for improving the training modules based on their experiences. Through this cyclical process, we aim to establish a fully student-led research group that cultivates student training, ultimately enhancing medical students’ research skills, experience, and productivity.

Research interest among medical students has been found to diminish as they advance through their academic years [15]. Our study supports these findings somewhat, as the majority of our cohort consisted of 19 preclinical students, compared with just 6 clinical students who were involved in clerkships and internships. To counteract this decline, efforts to promote research could be strengthened throughout their university education, potentially through the integration of CUREs. In addition, research indicates a decrease in the number of clinician-scientists in the United Kingdom, attributed partly to an inadequate influx of individuals into the “clinician-scientist pipeline” to replenish an aging workforce [16]. CUREs could potentially boost enthusiasm and familiarity with research, encouraging more individuals to pursue these career paths. Finally, although empirical evidence on the impact of student-led initiatives in academic medicine is limited, their widespread acceptance and popularity may suggest that students recognize a need for these programs and gain some value from participating in them [17]. Our study addresses this gap and serves as a call to action for policy makers.

### Limitations

As a pilot project, this research initiative revealed several areas needing revision and adjustment alongside the desired outcomes. First, the journal club’s membership was self-selected, which is likely to have influenced the project’s results. Participants were predisposed to be more motivated and interested in research, which may have increased engagement and contribution quality. This self-selection helped the project succeed by ensuring that participants were highly committed. However, it introduced a potential selection bias, reducing the generalizability of our findings. The predominance of motivated individuals may not accurately reflect the larger medical student population, particularly those who are less inclined or confident in conducting research. Moreover, while the publication of research papers by participants is an objective indicator of the project’s success, it is acknowledged that publication does not fully capture the breadth of research competencies sought by this program. Focusing solely on publication outcomes has the potential to overlook broader research skills such as ethical considerations, data management, and long-term research planning, all of which are critical to the sustainability of research practices. While we emphasized the importance of ethical considerations during our sessions, it is important to remember that the primary goal of our research was to help students take their first steps into the world of research. As a result, while we acknowledge the project’s scope and depth of limitations, it effectively served as an entry point for participants, many of whom had no prior research experience, to begin engaging with the research process.

Furthermore, various challenges surfaced during the project’s execution, leading to alterations in specific project elements and deadline extensions. One of these challenges was the inability to gain access to databases initially planned for original research study designs. Our requests were not met with a positive response, necessitating a change in the content and methodology of the projects requiring such access. For future iterations, we intend to include study proposals that leverage access to these databases. Another unexpected obstacle was the delay in obtaining ethical committee approvals for survey studies. This issue was not factored into the original timeline and, in light of this experience, we will allow for greater flexibility in project schedules moving forward. The project implementation process highlighted key factors that require consideration to ensure the project’s sustainability and ease of execution. One significant challenge was coordinating teamwork among participants with varied schedules. Sticking to the initial timeline was difficult for all participants, leading us to recommend gathering schedules beforehand and grouping students with similar availability together for future implementations. Decreased commitment from some participants was another issue. Over the course of the lengthy project, some students withdrew, primarily due to time constraints. This situation required the reassignment of certain tasks and additional courses for some students. Also, the project targeted a small, specific group of students and lacked a selection process. To address this, we propose the inclusion of an application process for more efficient training in future iterations. Another limitation was the lack of active engagement from participants in the question design. The tutors provided guidance and designed the research questions themselves as a starting point. Adding a course on this topic in future iterations could foster more active involvement from participants, thereby potentially improving project outcomes. Finally, dividing tasks among participants posed challenges in ensuring full research competency for all, as each participant focused on specific aspects of the project.

### Conclusions

This project effectively capitalized on the widespread accessibility of information to educate and enable students to partake in medical research, irrespective of their lack of direct hands-on experience. This approach carries significant weight as it equips students with the skills to draw data from preexisting studies, thereby exploiting the incremental nature of science. In addition, this method provides students from campuses with limited access to research facilities the opportunity to acquire experience in conducting a scientific project. Despite the encountered challenges, the project was successfully implemented, resulting in a notable advancement of the research skill set among previously inexperienced students. This translated into a demonstrable increase in undergraduate research output. The limitations identified during the project’s course provide a crucial understanding for improving future iterations of this initiative. Our goal is to perpetually refine and use this project as a supplement to traditional medical training, thus providing students with a keen interest in research the opportunity for self-paced learning and research training.

## Supplementary material

10.2196/51173 Multimedia Appendix 1 First module materials.

10.2196/51173 Multimedia Appendix 2 Second module materials.

## Abbreviations

CURE: course-based undergraduate research experience
