# Supplementary material for: A Pilot Project to Promote Research Competency in Medical Students Through Journal Clubs: Mixed Methods Study
Source: JMIR Med Educ. 2024 Oct 31;10:e51173. doi: 10.2196/51173 (PMC11542906; doi:10.2196/51173)
Supplement: Multimedia Appendix 1 [file mededu-v10-e51173-s001.docx]

**Multimedia Appendix 1:** First module materials.

The first module required the learners to watch the video series and complete the reading materials.

**Videos**

1. “Lecture Series: Using PubMed to Find High-Quality Literature”

<https://www.youtube.com/playlist?list=PLF2KCgTC6mbQX8Creoyl4jI8q-xeyfcyj>

1. “Learn how to use Zotero in 30 minutes”

<https://www.youtube.com/watch?v=BQL_7C-YqBk&t=1431s>

**Reading Materials**

1. Bramer WM, de Jonge GB, Rethlefsen ML, Mast F, Kleijnen J. A systematic approach to searching: an efficient and complete method to develop literature searches. *J Med Libr Assoc JMLA*. 2018;106(4):531-541. doi:10.5195/jmla.2018.283

2. McKeever L, Nguyen V, Peterson SJ, Gomez-Perez S, Braunschweig C. Demystifying the Search Button: A Comprehensive PubMed Search Strategy for Performing an Exhaustive Literature Review. *JPEN J Parenter Enteral Nutr*. 2015;39(6):622-635. doi:10.1177/0148607115593791

3. Ho GJ, Liew SM, Ng CJ, Hisham Shunmugam R, Glasziou P. Development of a Search Strategy for an Evidence Based Retrieval Service. *PloS One*. 2016;11(12):e0167170. doi:10.1371/journal.pone.0167170

4. Iskander JK, Wolicki SB, Leeb RT, Siegel PZ. Successful Scientific Writing and Publishing: A Step-by-Step Approach. *Prev Chronic Dis*. 2018;15:E79. doi:10.5888/pcd15.180085

5. Ecarnot F, Seronde MF, Chopard R, Schiele F, Meneveau N. Writing a scientific article: A step-by-step guide for beginners. *Eur Geriatr Med*. 2015;6(6):573-579. doi:10.1016/j.eurger.2015.08.005

6. Kliewer MA. Writing it up: a step-by-step guide to publication for beginning investigators. *J Nucl Med Technol*. 2006;34(1):53-59.
