# Supplementary material for: A Pilot Project to Promote Research Competency in Medical Students Through Journal Clubs: Mixed Methods Study
Source: JMIR Med Educ. 2024 Oct 31;10:e51173. doi: 10.2196/51173 (PMC11542906; doi:10.2196/51173)
Supplement: Multimedia Appendix 2 [file mededu-v10-e51173-s002.docx]

**Multimedia Appendix 2:** Second module materials.

The second module required learners to complete the verified online courses for their respective tasks in the project. The list of selected courses are provided below with the task title.

**Systematic Review**

1. Online course on Coursera: “Introduction to Systematic Review and Meta-Analysis” offered by Johns Hopkins University. (<https://www.coursera.org/learn/systematic-review>)

**Literature Review and Data Extraction**

1. Online course on Coursera: “Academic Information Seeking” offered by the University of Copenhagen and the Technical University of Denmark. (<https://www.coursera.org/learn/academicinfoseek>)

**Statistical Analysis**

1. Online course on Udemy: “SPSS for Research” offered by Bogdan Anastasiei. (<https://www.udemy.com/course/spss-for-research/>)

**Academic Writing**

1. Online courses on Coursera: “Writing in the Sciences” offered by Stanford University. (<https://www.coursera.org/learn/sciwrite>)
2. “How to Write and Publish a Scientific Paper (Project-Centered Course) offered by École Polytechnique. (<https://www.coursera.org/learn/how-to-write-a-scientific-paper>)
